# Identification of *Hyaloperonospora arabidopsidis* Transcript Sequences Expressed during Infection Reveals Isolate-Specific Effectors

**DOI:** 10.1371/journal.pone.0019328

**Published:** 2011-05-09

**Authors:** Adriana Cabral, Joost H. M. Stassen, Michael F. Seidl, Jaqueline Bautor, Jane E. Parker, Guido Van den Ackerveken

**Affiliations:** 1 Department of Plant-Microbe Interactions, Department of Biology, Utrecht University, Utrecht, The Netherlands; 2 Theoretical Biology and Bioinformatics, Department of Biology, Utrecht University, Utrecht, The Netherlands; 3 Centre for BioSystems Genomics (CBSG), Wageningen, The Netherlands; 4 Department of Plant-Microbe Interactions, Max Planck Institute for Plant Breeding Research, Cologne, Germany; University of Wisconsin-Milwaukee, United States of America

## Abstract

Biotrophic plant pathogens secrete effector proteins that are important for infection of the host. The aim of this study was to identify effectors of the downy mildew pathogen *Hyaloperonospora arabidopsidis* (*Hpa*) that are expressed during infection of its natural host *Arabidopsis thaliana*. Infection-related transcripts were identified from Expressed Sequence Tags (ESTs) derived from leaves of the susceptible Arabidopsis Ws *eds1-1* mutant inoculated with the highly virulent *Hpa* isolate Waco9. Assembly of 6364 ESTs yielded 3729 unigenes, of which 2164 were *Hpa*-derived. From the translated *Hpa* unigenes, 198 predicted secreted proteins were identified. Of these, 75 were found to be *Hpa*-specific and six isolate Waco9-specific. Among 42 putative effectors identified there were three Elicitin-like proteins, 16 Cysteine-rich proteins and 18 host-translocated RXLR effectors. Sequencing of alleles in different *Hpa* isolates revealed that five *RXLR* genes show signatures of diversifying selection. Thus, EST analysis of *Hpa*-infected Arabidopsis is proving to be a powerful method for identifying pathogen effector candidates expressed during infection. Delivery of the Waco9-specific protein RXLR29 *in planta* revealed that this effector can suppress PAMP-triggered immunity and enhance disease susceptibility. We propose that differences in host colonization can be conditioned by isolate-specific effectors.

## Introduction

Plant pathogens secrete an arsenal of effector molecules that modulate host responses to enable successful infection. Effector proteins constitute part of the secretome of the invading organism and are regarded as being crucial for pathogenicity [1]. Pathogen-derived effectors target different sites in host plant tissues. While apoplastic effectors are secreted into the plant extracellular space, host-translocated effectors are delivered into host cells after secretion from the pathogen [2], [3], [4]. For successful colonization, several layers of defense have to be overcome by the pathogen. An initial barrier is conferred by host membrane-resident receptors recognizing pathogen-associated molecular patterns (PAMPs), molecules that are structurally conserved among related pathogenic microorganisms and not present in the host [5], [6], [7]. PAMP-triggered immunity (PTI) is a resistance response that generally protects plants against a broad range of non-adapted microorganisms. Several bacterial effector proteins delivered to host cells by the type III secretion system (TTSS) have been shown to suppress PTI [8], [9]. A second layer of defense (effector-triggered immunity, ETI) can be activated through recognition of particular pathogen effectors or their actions on host targets by Resistance (R) proteins. ETI is a more acute plant reaction often involving programmed cell death at infection sites. Effectors can modulate the ETI response or mutate to circumvent recognition resulting in a co-evolutionary battle in which the pathogen attempts to evade host resistance and new plant *R* genes evolve to restrict further pathogen growth [10], [11]. Therefore, pathogen effectors with high rates of gene loss, duplication or diversification are likely to be elicitors and/or modulators of plant immunity depending on the host genetic background they encounter [12], [13].

Some of the most highly co-evolved interactions are between plants and biotrophic pathogens. At one end of the biotroph spectrum are hemi-biotrophic species that initially colonize living cells but then switch to necrotrophy. At the other end are obligate biotrophs that maintain host cell integrity and depend entirely on their host for growth and completion of their life cycle. Obligate biotrophs have evolved sophisticated mechanisms for host cell manipulation and defense suppression [14]. Characterizing the activities and targets of biotroph-secreted effectors should therefore provide insights to how host-adapted pathogens promote disease and avoid recognition.

The obligate biotroph *Hyaloperonospora arabidopsidis* (*Hpa*) naturally infects the model plant *Arabidopsis thaliana*, causing downy mildew disease [15]. *Hpa* is a highly specialized oomycete pathogen with a narrow host range. Analysis of the Arabidopsis-*Hpa* interaction has been particularly informative because of the extensive genetic variation in responses of different Arabidopsis accessions to a correspondingly diverse set of natural pathogen isolates [16], [17]. Strong differences in resistance of Arabidopsis accessions to particular *Hpa* isolates were found to be conferred by *R* genes often residing at polymorphic loci [18], [19], [20], [21]. Although plant-infecting oomycetes exhibit a fungal-like morphology and feeding structures (haustoria), they form a phylogenetically distinct group of eukaryotic pathogens that, together with brown algae and diatoms, belong to the Stramenopile lineage (heterokonts) [22]. The genomes of several agronomically important hemi-biotrophic oomycete pathogen species have been sequenced, such as *Phytophthora sojae* (causing soybean root and stem rot), *P. ramorum* (sudden oak death) and *P. infestans* (potato late blight) [23], [24]. Recently, the genome sequence of the *Hpa* isolate Emoy2 has also become available [25]. Extensive oomycete genome sequence information combined with data from expression profiling of pathogen and host genes at different infection stages is now serving as a basis to identify potentially important pathogen effector genes [26], [27], [28]. To date, a number of oomycete effector proteins triggering ETI have been identified [11], [29], [30], [31], [32], [33]. Notably, these molecules are characterized by a secretion signal followed by a conserved “RXLR” motif (where X can be any amino acid residue) and often a stretch of acidic amino acids ending with the motif “EER”. Whereas the RXLR motif is required for effector translocation into the host cell, the C-terminal region comprises a more variable “effector domain” [34], [35], [36], [37]. ATR1 and ATR13 are two RXLR type effectors of *Hpa* that are recognized by Arabidopsis *R* genes [11], [30]. High levels of *ATR1* and *ATR13* polymorphism among *Hpa* isolates suggest that diversifying selection has driven the evolution of both genes [11], [13], [30].

Previous studies aimed at identifying *Hpa* pathogenicity genes by isolating pathogen transcripts expressed during infection have so far resulted in the isolation of only a few candidate effectors. A differential cDNA-amplified fragment length polymorphism (AFLP) analysis was used by van der Biezen et al. [38] on *Hpa*-infected Arabidopsis leaves leading to identification of 10 *Hpa*-derived cDNA fragments. In another study, a suppression subtractive hybridization (SSH) strategy was used to identify 25 *Hpa*-expressed genes from infected Arabidopsis [39].

Here we report a transcript sequencing approach based on Expressed Sequence Tag (EST) analysis of *Hpa*-infected Arabidopsis leaves. To increase the proportion of *Hpa* transcripts in a mixed sample of plant and pathogen RNA we used a highly virulent *Hpa* isolate (Waco9) to infect a hyper-susceptible Arabidopsis mutant (*eds1*) [40] and collected tissues at a time point of maximum hyphal and haustorial growth. From a set of 2164 *Hpa* unigenes we have identified and classified *Hpa*-secreted proteins into several effector categories based on the presence of characteristic domains/motifs. Eighteen of the *Hpa* proteins belong to the class of host-targeted RXLR effectors. While several effector candidates are shared with other *Hpa* isolates, and in some cases oomycete species, six predicted effector proteins from Waco9 are not detected in the genome of the sequenced isolate Emoy2, highlighting the dynamic nature of *Hpa* pathogenicity.

## Results

### EST sequencing of *Hpa*-infected Arabidopsis tissue

**Figure 1:**
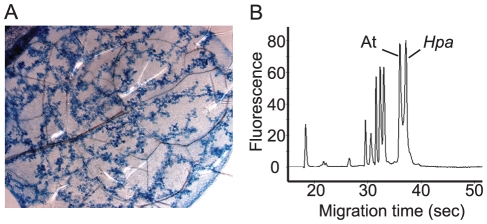
*Hpa* isolateWaco9-infected Arabidopsis seedlings used for cDNA library construction. (A) Trypan blue staining of infected Arabidopsis leaves (Ws *eds1-1*) showing extensive hyphal growth in the absence of asexual sporulation at 5 dpi. (B) Bioanalyzer profile of total RNA obtained from *Hpa* Waco9-infected leaf material. The 28S rRNA peaks of Arabidopsis (At) and Waco9 (*Hpa*) are indicated.

*Hpa* isolates Emoy2, Emwa1, Noco2 and Waco9 were assessed for growth on the Arabidopsis *enhanced disease susceptibility 1* mutant Ws *eds1-1* [40]. Microscopic analysis of infected leaves after trypan blue staining revealed the extent of pathogen growth. A higher level of colonization of Arabidopsis leaves, with abundant hyphal growth and haustoria projections formed in adjacent plant cells, was observed for *Hpa* isolate Waco9 compared to Emoy2 (Fig. S1) and other isolates tested (results not shown). Waco9-infected leaves harvested at 5 dpi (before sporulation) (Fig.1A) were therefore used for the construction of a cDNA library.

Total RNA isolated from the Waco9-infected material revealed ribosomal RNA (rRNA) peaks of *Hpa* and Arabidopsis (Fig. 1B). The observed peaks were validated as rRNA originating from *Hpa* or Arabidopsis by comparing profiles of infected leaves with those of RNA from *Hpa* conidiospores mixed in different proportions with Arabidopsis leaf RNA (Fig. S2). This analysis showed that ∼50% of RNA extracted from the Waco9-infected leaves was derived from *Hpa*. Poly (A^+^) RNA was isolated from the infected leaf total RNA preparations and size-fractionated cDNAs ranging from 500 to 5000 bp were used for cDNA library construction.

**Figure 2:**
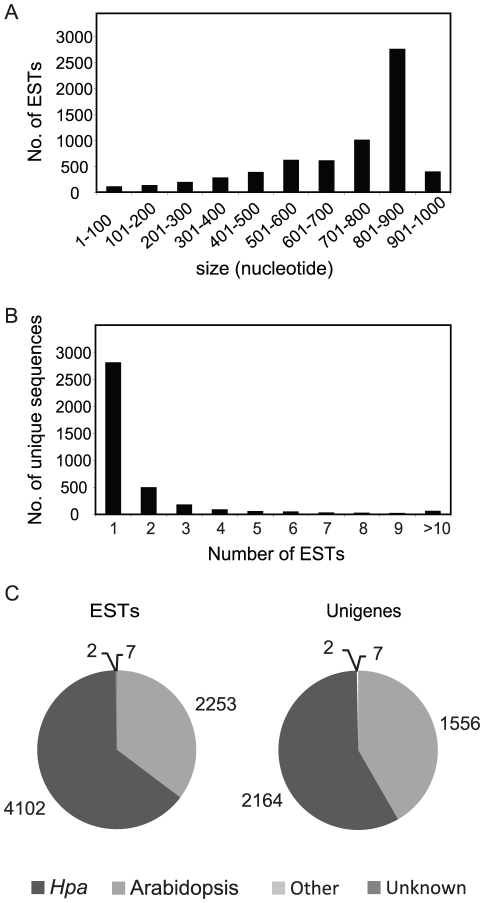
ESTs and unigenes derived from *Hpa* Waco9 infections. (A) Size distribution of the 6364 ESTs (in nucleotides). (B) Distribution of 3729 unique sequences based on the number of assembled ESTs. (C) Distribution of 6364 ESTs and 3729 unigenes based on sequence origin. Numbers of sequences with significant similarity to Arabidopsis, *Hpa*, other oomycetes, other organisms and unknown, are indicated.

7680 sequencing reactions corresponding to the 5′ end of the cDNAs were performed. After vector trimming and removal of low quality sequences and chimeras, 6364 ESTs remained ranging in length from 50 to 1000 nucleotides (nt). The majority (84%; 5321 sequences) of ESTs had a read length of more than 500 nt (Fig. 2A). Assembly of ESTs yielded a total of 3729 unique sequences (unigenes) consisting of 2810 (∼75%) singletons and 919 (∼25%) contigs with two or more ESTs (Fig. 2B).

To define the origin of the unigene sequences, BLASTN searches were performed against the genome sequences of Arabidopsis, *Hpa*, three oomycete pathogens (*P. infestans*, *P. sojae* and *P. ramorum*) and the NCBI nr nucleotide database. Almost all unigenes (3722; ∼99.8%) showed significant similarity (E<10^−5^) to known sequences. Whereas 1556 unigenes (∼42%, corresponding to 2253 ESTs) were identified as Arabidopsis sequences, 2164 unigenes (∼58%; 4102 ESTs) had homologues within the sequenced *Hpa* isolate Emoy2 (Fig. 2C). Of the remaining unigenes two had high identity scores with sequences of other non-plant and non-oomycete organisms and seven did not show significant homology to any sequence in the databases (unknown sequences in Fig. 2C), nor contained a signal peptide in the predicted open reading frame and were therefore not further analyzed. Altogether, we defined 2164 unigenes as *Hpa*-derived to use them for subsequently functional classification.

### Identification of putative secreted *Hpa* proteins

Secreted proteins of fungal and oomycete plant pathogens have been shown to function as pathogenicity factors [4], [41], [42]. We therefore performed a comprehensive search for *Hpa* transcripts encoding secreted proteins. From the 2164 *Hpa* unigenes a set of 198 unigenes (822 ESTs, 9.15% of unigenes) encoded proteins with a predicted signal peptide as analyzed with SignalP software [43], [44], and without putative transmembrane domains. The EST-derived unigenes are significantly enriched (p = 4e^−6^) for transcripts encoding predicted secreted proteins when compared to the genome wide percentage of 6.8% (Table S2).

**Figure 3:**
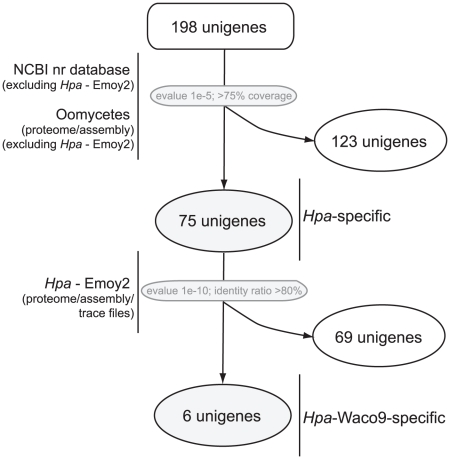
Scheme showing categorization of *Hpa* Waco9 unigenes encoding predicted secreted proteins. Sequences with homologues in oomycetes (excluding *Hpa*) and other organisms are grouped (123 sequences). The remaining 75 unigenes were used to search for homologues in the assembled Emoy2 genome and trace files. A set of six unigenes with no significant similarity to any other sequence is defined as Waco9-specific.

To define which of the *Hpa* secreted proteins are shared with other organisms, the 198 unigenes were compared to the genome sequences of three oomycete pathogens (*Phytophthora infestans*, *P. sojae*, and *P. ramorum*) and sequences in GenBank, excluding *Hpa*. Significant blast hits (E<10^−5^ and sequence coverage >75%) were obtained for 123 unigenes (Fig. 3). The remaining 75 sequences (38%) did not have a significant hit with other oomycetes or non-*Hpa* sequences in GenBank, suggesting that these genes are *Hpa*-specific. As the *Hpa* genome sequence is derived from isolate Emoy2 [25] and the ESTs isolated here are from isolate Waco9, we searched for possible differences between these two isolates. The 75 *Hpa*-specific unigenes were queried against the assembled Emoy2 genome sequence and manually compared to *Hpa* trace files (http://www.ncbi.nlm.nih.gov/BLAST) to search for sequences that might be missing from the *Hpa* genome assembly. No significant hits were obtained for six sequences, suggesting that these unigenes are present in *Hpa* isolate Waco9 but not in Emoy2.

A functional annotation of the 198 *Hpa* unigenes predicted to encode secreted proteins was performed by comparison to the Pfam database of protein domains [45] and by manual annotation to search for specific classes of effectors. No function could be defined for 108 sequences that were therefore classified as unknowns. Table S3 shows the putative functions or domains assigned to 90 unigene-encoded proteins. A selection of categories potentially associated with pathogenicity (adapted from [23]), and the number of ESTs and unigenes belonging to each group, is shown in Table 1. Unigenes encoding putative pathogenicity proteins (58 sequences) were classified into three categories: hydrolase enzymes, protection against oxidative stress, and effectors. These 58 unigenes, together with the 108 sequences with unknown function, were then divided according to the group of organisms in which homologues were identified. From the 108 unknown sequences, 51 are categorized as *Hpa*-specific (Table 1). Homologues in other oomycetes were identified for all unigenes predicted to function as hydrolases or in protection against oxidative stress (Table 1), indicating that proteins in these classes are conserved among oomycetes. We looked for various families of effectors that are associated with the triggering or manipulation of host cell defenses [46], [47], [48]. Putative effector proteins formed the largest functional group identified, comprising 42 unigenes. In this category we assigned 16 proteins as Cysteine-rich (CR) proteins and 18 as belonging to the RXLR family. The majority of both effector types (23 out of 34 unigenes) appear to be *Hpa*-specific since no related sequences were detected in other oomycetes. Moreover, two CR proteins and four RXLRs were not identified in the Emoy2 genome suggesting they are isolate Waco9-specific. Other classes of predicted effector proteins such as Necrosis-inducing like proteins, elicitins, Crinklers and cyclophilin had homologues within other oomycetes, suggesting that these sequences belong to a common core of putative effectors present in oomycete organisms. The Crinkler identified carries a variation of the LxLFLAK translocation motif (Fig. S3), which has also been described to be present in several Crinklers of *P. infestans* (Haas et al 2009).

**Table 1:** Classification of predicted *Hpa* secreted proteins.

		Total					
		ESTs	Unigenes	Other Oomycetes	*Hpa-*specific	Emoy2	Waco9-specific
Hydrolases		**41**	**9**	**9**	**0**	**0**	**0**
	Cell Wall Degrading Enzymes	32	4	4	0	0	0
	Serine proteases	5	1	1	0	0	0
	Serine carboxypeptidases	2	2	2	0	0	0
	Aspartyl proteinase/proteases	1	1	1	0	0	0
	Cysteine protease/proteinases	1	1	1	0	0	0
Protection against oxidative stress		**35**	**7**	**7**	**0**	**0**	**0**
	Glutathione s-transferases	1	1	1	0	0	0
	Reductases	16	2	2	0	0	0
	Glutaredoxins	3	1	1	0	0	0
	Thioredoxins	15	3	3	0	0	0
Effectors		**342**	**42**	**17**	**24**	**18**	**6**
	RXLRs	40	18	4	14	10	4
	Cysteine-rich proteins	279	16*1	6	9	7	2
	Elicitins	9	4	3	1 *2	1	0
	Necrosis-inducing like proteins	3	2	2	0	0	0
	Crinklers	1	1	1	0	0	0
	Cyclophilins	2	1	1	0	0	0
unknowns		361	108	55	51	51	0

The distribution in different pathogenicity categories and organisms in which homologues were identified are shown. Functional classification is based on Pfam searches and manual annotation.

*1 One predicted Cysteine-rich candidate has homology to *Tetrahymena thermophila* and to *Hpa* sequences. No homologue was found in other oomycetes.

*2 Elicitin HaELL2 is classified as *Hpa*-specific due to its extended specific C-terminal domain.

### Elicitin signatures in *Hpa*

**Figure 4:**
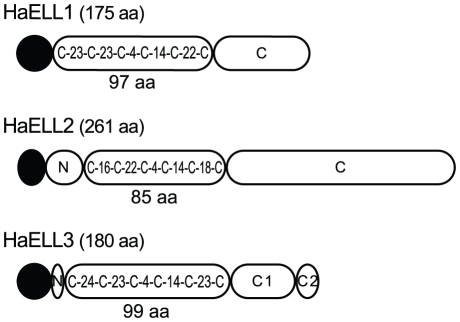
Schematic representation of protein domains of three identified *Hpa* HaELLs. The predicted signal peptide is shown in black. A region between the signal peptide and the elicitin domain found in HaELL2 and 3, with respectively 23 and 8 amino acids, is shown (N). The pattern of cysteine spacing and the size of the elicitin domain are depicted. The percentage of highly abundant amino acid residues in the C-terminus is: HaELL1: T: 14%, A: 12%, S: 7%; HaELL2: T: 28%, A: 13%, S: 10% per domain is depicted. The C-terminus of HaELL3 is divided into two regions (C1 and C2). C1: S: 51%, T: 19%; C2: D;56%, E:31%.

Elicitins (ELIs) are extracellular effectors characterized by a 98 amino acid conserved domain with a core of six cysteines in a specific spacing pattern allowing their classification into different groups. Elicitin-like proteins (ELLs) have more variation in the size and sequence of their elicitin domains [49]. Although Pfam searches identified four ELL unigenes in Waco9 (Table 1), only three of these sequences were further analyzed (Fig. 4) since the fourth contained only five cysteines and therefore did not classify as an ELI/ELL. The HaELL1, 2 and 3 encoded proteins have fewer than 5% cysteine residues and are classified as alpha-elicitins due to their acidic pI [50]. The different size and cysteine spacing in the elicitin domain of these three HaELLs is shown in Fig. 4. While HaELL2 resembles *Phytophthora* ELL-1 proteins [49], the Cys pattern in HaELL1 and 3 appears to be specific for *Hpa*. All three proteins have an extended C-terminal region following the elicitin domain, a feature of other oomycete ELLs [49]. This region is smaller in HaELL1 and 3 (57 and 53 amino acids, respectively) in comparison to HaELL2 (137 amino acids). The C-terminal domain appears to have a biased amino acid composition as it is rich in threonine, alanine and serine (Fig. 4). The high abundance of threonine and serine residues in the C-terminal part of HaELL1, 2 and 3 points to numerous potential sites for O-glycosylation (predicted by NetOGlyc 3.1) that could link the proteins to the cell wall. A hydrophobic region containing a GPI anchor was predicted in the C-terminal part of HaELL2, suggesting that this protein might be anchored to the plasma membrane. O-glycosylation sites and GPI anchor regions have been described for other *Phytophthora* ELIs/ELLs [49].

### *Hpa* derived Cysteine-rich (CR) proteins

**Figure 5:**
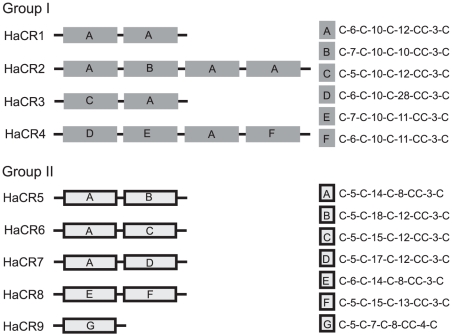
Two groups of HaCR proteins identified with distinct cysteine spacing patterns. The boxes show the pattern repeated in each protein.

A number of CR proteins secreted by fungal pathogens have been shown to be recognized by R proteins in resistant host plants [51], [52], [53]. The cysteines in these proteins form disulphide bridges that contribute to their structural stability in the plant apoplast, an environment rich in proteases. To identify HaCR candidates among the putative *Hpa* secreted proteins, we determined the relative number of cysteine residues in the full-length translated sequences. A total of 16 HaCR proteins were identified which contain more than 5% cysteines and range in size from 72 to 405 amino acids (Table 2). Nine of the HaCR candidates could be placed into two groups based on the pattern and spacing of the cysteines (Fig. 5). The cysteine motif in group I and II is repeated one or more times in most group members. As shown in Tables 1 and 2, six HaCR candidates have homologues in other oomycete pathogens. Candidates from Groups I and II do not have an obvious oomycete counterpart. However for HaCR2 significant homology (blast E value <1e^−5^) was found to a protein of the ciliate *Tetrahymena thermophila*.

**Table 2:** Characteristics of CR effectors identified in the set of *Hpa* secreted proteins.

Group	Name	ESTs	Homologues*	Identity Emoy2	size (amino acids)	% Cys
I	HaCR1	84	*Hpa-*specific	98%	127	9
I	HaCR2	21	*T. thermophila*	99%	216	11
I	HaCR3	40	*Hpa*-specific	97%	128	9
I	HaCR4	1	*Hpa*-specific	100%	236	10
II	HaCR5	18	*Hpa*-specific	96%	116	11
II	HaCR6	37	Waco9	not in Emoy2	114	11
II	HaCR7	18	*Hpa*-specific	89%	116	11
II	HaCR8	3	*Hpa*-specific	96%	112	11
II	HaCR9	36	*Hpa*-specific	100%	72	8
other	HaCR10	7	Oomycetes	100%	205	8
	HaCR11	1	Oomycetes	100%	120	13
	HaCR12	2	Oomycetes	100%	96	9
	HaCR13	3	Oomycetes	99%	278	6
	HaCR14	2	Oomycetes	99%	405	7
	HaCR15	2	Oomycetes	100%	117	5
	HaCR16	4	Waco9/Maks9	not in Emoy2	74	11

*Homologues were denoted according to the organisms in which significant blast hits were identified. Oomycetes: significant blast hits obtained only with oomycete sequences; *Hpa*-specific: blast hits obtained only with the Emoy2 genome. If no significant blast hit was obtained with Emoy2 or any other organism, then the sequence is defined as a Waco9.

Notably, the HaCR sequences comprised the largest number of ESTs among the selected pathogenicity factors (Table 1), indicating that this class of effectors is highly expressed during infection. Blast searches against the Emoy2 genome identified homologues of the Waco9 HaCRs (Table 2). Six proteins (HaCR4, 9, 10, 11, 12 and 15) were absolutely conserved (100% identity) between the two isolates. The most divergent protein (HaCR7) had 89% identity between isolates Waco9 and Emoy2. No Emoy2 homologues were found for HaCR6 and HaCR16, the latter being a homologue of the previously described Ppat23 protein of isolate Maks9 [39]. HaCR1, represented by the highest number of ESTs (84) among the secreted proteins, is a homologue of Ppat14 from *Hpa* isolate Maks9 [39].

### Classification of host-translocated *Hpa* RXLR proteins

RXLR proteins comprise a class of oomycete effectors that are translocated into the plant cell [36]. All known oomycete effector proteins that are recognized by specific plant R proteins belong to this class [11], [29], [30], [31], [32], [33]. The *Hpa* unigenes encoding putative secreted proteins were therefore mined for candidate RXLR effectors. Proteins containing either an RXLR or RXLQ/RXLG motif in the mature protein were selected. Variations in the last arginine of the RXLR still allows protein translocation into the host cell [37]. We identified 18 RXLR candidates (including one RXLQ containing protein) encoding relatively small proteins ranging from 115 to 340 amino acids (Table 3). The distance between the RXLR/Q motif and the signal peptide cleavage site varied from 15 to 51 amino acids indicating that the RXLR/Q motif is near the N-terminus. The presence of an acidic region (EER) downstream of the RXLR motif was identified in eight RXLR candidates (Table 3) and two (RXLR3 and RXLR6) have a putative nuclear localization signal as predicted by Psort.

**Table 3:** Sequence features of candidate Waco9 RXLR effector proteins.

Name	*Hpa* RXLR gene IDs (1)	ESTs	size (amino acids)	RXLR distance (2)	EER (3)	Homologues (4)
RXLR3	-	1	129	29	-	*Hpa*-specific
RXLR4	-	3	134	29	-	Waco9
RXLR5	-	1	340	30	-	Oomycetes
RXLR6	HaRXL80	1	129	27	+(44)	*Hpa*-specific
RXLR7	HaRXL17	1	305	20	+(32)	*Hpa*-specific
RXLR9	HaRXL78	2	150	28	-	*Hpa*-specific
RXLR12	-	3	125	28	-	Waco9
RXLR13	HaRXL76	5	286	32	+(49)	*Hpa*-specific
RXLR15	HaRXL77	3	129	15	+(37)	Oomycetes
RXLR16	HaRXL30; HaRXL79	3	198	28	+(41)	Waco9
RXLR17 (RXLQ)	HaRXL42	2	135	28	+(41)	*Hpa*-specific
RXLR18	-	2	209	51	-	Oomycetes
RXLR19	-	1	299	31	-	*Hpa*-specific
RXLR20	HaRXL10	1	241	23	-	*Hpa*-specific
RXLR21	HaRXL37; HaRXL75	1	115	29	+(44)	Oomycetes
RXLR22	-	1	137	29	-	Waco9
RXLR23	HaRXL4	5	304	32	+(49)	*Hpa*-specific
RXLR29	-	4	132	28	-	*Hpa*-specific

**Figure 6:**
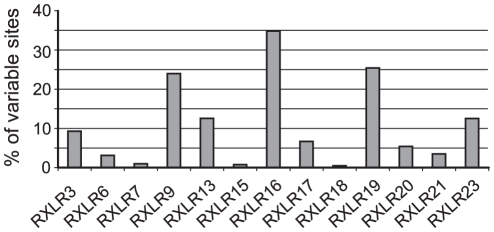
Polymorphic *Hpa* RXLR proteins. Numbers of variable sites were determined using Mega4 for RXLR proteins possessing two or more protein variants.

(1) Gene IDs refer to RXLR sequences identified in Emoy2 [25].

(2) Distance to signal peptide cleavage site.

(3) In brackets the distance to signal peptide cleavage site.

(4) Homologues found based on blast searches as described for figure 6.

From the 18 RXLR sequences identified here, only four proteins have homology with other oomycetes, indicating that the majority of the RXLR candidates are *Hpa*-specific (Tables 1 and 3). Among these four RXLR proteins, RXLR5 and 18 showed a high degree of similarity with homologues in *P. infestans* (75% and 72% identity, respectively, with 100% sequence coverage). From the 14 *Hpa*-specific proteins, blast searches revealed that 10 RXLRs have corresponding genes in Emoy2 (Tables 1 and 3). Further analysis of these sequences revealed that RXLR29 is represented by a null allele in *Hpa* Emoy2 caused by a frame-shift mutation resulting in a premature stop codon (Fig. S4). For RXLR4, 12, 16 and 22 no significant blast hits with the Emoy2 genome sequence were obtained (Tables 1 and 3). It is possible that these *RXLR* genes are either highly polymorphic or absent from the Emoy2 genome.

### Allelic diversity of RXLR effector candidate genes

Coding sequences of the 18 RXLR candidate effectors were deduced from seven *Hpa* isolates. Table 4 shows the number of protein variants found for each RXLR and their distribution among the different *Hpa* isolates. For example, RXLR3 is represented by two allelic forms: Waco9, Noks1, Emco5 and Cala2 share variant A, Emoy2 and Maks9 carry protein variant B and Hind2 has no *RXLR3* gene. Heterozygosity was also observed for some RXLRs (e.g. RXLR6 in Cala2 contains both protein variants A and B). *RXLR5* was the least variable gene with identical proteins in all seven *Hpa* isolates.

**Table 4:** Allelic variants identified for 18 *Hpa* RXLR candidate proteins and their distribution among 7 *Hpa* isolates.

		Allele at the indicated *Hpa* isolate						
	No. Alleles	Cala2	Emco5	Emoy2	Hind2	Maks9	Noks1	Waco9
RXLR3	2	A	A	B	-	B	A	A
RXLR4	1	-	-	-	A	-	-	A
RXLR5	1	A	A	A	A	A	A	A
RXLR6	4	A,B	C	B	D	C	C	A
RXLR7	3	B	C	B	B	B	B	A
RXLR9	5	B;C	E	D	B	E	D	A
RXLR12	1	-	-	-	-	-	-	A
RXLR13	6	B	A	D	E	F	C	A
RXLR15	2	B	B	A;B	B	A	B	A
RXLR16	6	B	C	B;C	D	E	F	A
RXLR17	2	B	B	B	B	B	B	A
RXLR18	2	B	A	A	A	A	B	A
RXLR19	5	B	C	D	E	B	D	A
RXLR20	4	-	A;B	A	C	C	D	A
RXLR21	3	A	A	A;B	C	B	B	A
RXLR22	1	-	-	-	-	-	-	A
RXLR23	6	C	D	D	E	A;B	F	A
RXLR29	3	B	B	B	B	B;C	B	A

The letters indicate protein variants present at each isolate (protein level). When 2 letters are present it means that the *Hpa* isolate is heterozygous for that locus and has one copy of each allele.

(-) indicates that no allele was obtained.

No allele sequences were amplified for *RXLR12* and *22* in Emoy2 or in any of the five other *Hpa* isolates, indicating that these are Waco9-specific. *RXLR4* was only amplified from isolates Hind2 and Waco9. Although blast searches did not reveal a significant hit for RXLR16 in the Emoy2 genome (Table 3), allele sequences were obtained for Emoy2 as well as the other isolates. Due to a high level of polymorphism between the RXLR16 protein sequences in Waco9 and Emoy2, the blast hit obtained was excluded based on cut-off values.

The *RXLR29* gene was of particular interest because Waco9 is the only isolate of these analyzed that contains an intact full-length ORF. In the remaining six *Hpa* isolates, *RXLR29* displayed insertions/deletions giving rise to frame shifts resulting in null alleles (Fig. S4). In Maks9 we also found two different truncated versions of RXLR29. These results show that a functional RXLR29 protein is only present in Waco9 suggesting that it has been counter-selected in the other *Hpa* isolates examined.

Of the 13 RXLR candidate effectors with at least two different protein variants, we defined the percentage of variable sites within the protein sequences (Fig. 6). RXLR9, 13, 16, 19 and 23 are the most polymorphic effector proteins with more than 10% variable sites among the isolates sequenced. The ratio of >1 non-synonymous (dN) to synonymous (dS) nucleotide substitutions [54] in RXLR 9, 13, 16, 19 and 23 suggests that these genes are under positive selection to maintain amino acid diversity (Table 5).

**Table 5:** Ratio of non-synonymous (dN) and synonymous (dS) substitutions of *RXLR* genes.

Gene	No seq.	dN	dS	dN/dS	Z-statistic	P-value
RXLR3	6	0,026	0,011	2,4	1,6	0,06
RXLR4	2	0	0	-	-	-
RXLR5	7	0	0,001	-	-	-
RXLR6	8	0,006	0,007	0,9	-0,3	1
RXLR7	7	0,0012	0	-	-	-
**RXLR9**	**8**	**0,071**	**0,023**	**3,1**	**3,5**	**0,0004**
**RXLR13**	**7**	**0,025**	**0,006**	**4,2**	**3,9**	**0,0001**
RXLR15	8	0,002	0	-	-	-
**RXLR16**	**8**	**0,103**	**0,041**	**2,5**	**4,8**	**0,000002**
RXLR17	7	0,008	0,003	2,7	1,3	0,103
RXLR18	8	0,001	0,003	0,3	-0,8	1
**RXLR19**	**7**	**0,055**	**0,03**	**1,8**	**2,7**	**0,004**
RXLR20	8	0,007	0,013	0,5	-0,9	1
RXLR21	8	0,009	0,012	0,8	-0,3	1
**RXLR23**	**8**	**0,025**	**0,007**	**3,6**	**3,2**	**0,001**
RXLR29	8	0,03	0,021	1,4	0,9	0,19

Nucleotide sequences obtained for each RXLR candidate of *Hpa* isolates were used to calculate the ratio dN/dS. Genes under positive selection exhibit a dN/dS ratio higher than 1 and a P-value <0.005 (in bold).

### *Hpa* RXLR29 suppresses pathogen-induced callose deposition

**Figure 7:**
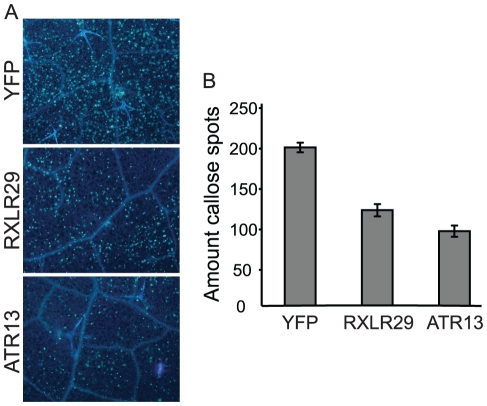
RXLR29 suppresses callose deposition in bacteria-inoculated Arabidopsis leaves. Five-week-old Col-0 leaves were hand-infiltrated with 1×10^8^ cfu/ml *Pst* DC3000ΔCEL carrying YFP, RXLR29 or ATR13, as indicated. Leaf samples were taken at 12 h after infection and stained with aniline blue to visualize callose (A). The number of callose spots was quantified in each sample (B). The experiment was repeated three times with similar results.

Since only isolate Waco9 appears to have retained a functional RXLR29 protein, we selected this gene to determine a potential effector activity. We used a bacterial effector delivery vector (EDV) system which was previously shown to successfully deliver the *Hpa* effector ATR13 to Arabidopsis leaf cells by fusion to the N-terminal portion of a TTSS bacterial effector [42]. *RXLR29* was cloned in the EDV system and expressed in the *Pseudomonas syringae* pv *tomato* (*Pst*) DC3000ΔCEL mutant strain which lacks the conserved effector locus (CEL) and is therefore unable to efficiently suppress PTI [55], [56]. Pathogen-induced deposition of callose at the cell wall is used as a marker of PTI [57]. We therefore measured whether RXLR29 delivery affects the capacity of *Pst* DC3000ΔCEL to induce callose deposition after infiltrating bacteria into leaves. In these experiments, YFP and ATR13 were expressed by the EDV vector in *Pst* DC3000ΔCEL, respectively as negative and positive controls. Arabidopsis Col-0 exhibited numerous callose deposits after infiltration with *Pst* DC3000ΔCEL-YFP (Fig. 7 A, B). The extent of callose deposition was reduced upon delivery of ATR13 and RXLR29 compared to YFP, indicating that both effectors suppress this form of PAMP-triggered cell wall defense.

### *Hpa* RXLR29 enhances disease susceptibility *in planta*

**Figure 8:**
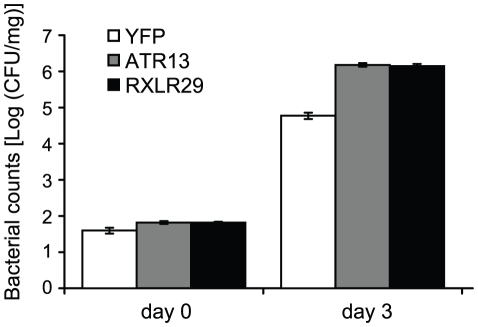
RXLR29 enhances bacterial growth in Arabidopsis leaves. Five-week-old Col-0 leaves were hand-infiltrated with 5×10^5^ cfu/ml *Pst* DC3000-LUX delivering YFP, ATR13 or RXLR29, as indicated. Bacterial growth in leaves at 0 and 3 dpi was measured by number of colony forming units (cfu) per mg of leaf tissue. Error bars indicate the standard error of bacterial counts. Enhanced growth was observed at day 3 for *Pst* delivering ATR13 or RXLR29 compared to YFP (T-test p value <0.0005).

Suppression of defense can lead to enhanced susceptibility to pathogen infection. We therefore examined if EDV-delivery of RXLR29 affects the growth of *Pseudomonas* bacteria. ATR13 enhances *Pst* DC3000 growth on Arabidopsis [42] and was used as positive control. We found that delivery of RXLR29 or ATR13 enhanced growth of *Pst* DC3000 compared to delivery of YFP (Fig. 8). Also, the RXLR29 and ATR13 delivering bacteria produced more chlorosis in Col-0 at 3 dpi than the control (YFP). The enhanced level of bacterial growth was comparable between delivery of ATR13 and RXLR29. The Waco9 RXLR29 protein is therefore likely to be a bona fide *Hpa* effector.

## Discussion

In this paper we described the identification of effector protein transcripts from the obligate biotrophic pathogen *Hyaloperonospora arabidopsidis* by analyzing 6364 ESTs of densely-infected leaf material from the hyper-susceptible Arabidopsis mutant Ws *eds1-1*. Comparison of different isolates of *Hpa* showed large differences in infection severity. Isolate Waco9 was chosen for sequencing as it reproducibly displayed abundant colonization of Arabidopsis leaves. To further increase *Hpa* biomass in infected leaves, growth conditions were adapted by reducing the relative humidity (see Materials and Methods) to delay pathogen sporulation. We were thus able to generate *Hpa*-infected plant material containing equal amounts of *Hpa* and Arabidopsis rRNA (Fig. 1C). The available genome sequences of Arabidopsis and *Hpa* were used to trace the origins of the ESTs. Surprisingly, the number of ESTs derived from *Hpa* was almost twice as high as from Arabidopsis indicating high relative amounts of *Hpa* mRNA in the infected leaf tissues.

A total of 3729 unigenes were assembled consisting of 2164 *Hpa* and 1556 Arabidopsis sequences. About 9% of the *Hpa* unigenes (198 sequences) encoded predicted secreted proteins that are likely targeted to the host-pathogen interface and might therefore play a role in the infection process. Previous analysis of EST libraries generated from four developmental stages of *P. sojae* revealed the highest frequency of ESTs encoding secreted proteins in interaction libraries [27].

From the 198 *Hpa* secreted proteins, a putative function could be assigned to 90 sequences based on the presence of known domains/motifs, specific features (e.g. relative number of cysteine residues) or similarity to known proteins (Table S3). A set of 58 sequences comprising potential pathogenicity factors was further classified into different categories of which the largest group (42 unigenes) corresponded to putative effector proteins (Table 1). EST analysis has been shown to be an effective method to identify pathogen genes encoding effector proteins. For example, Crinklers were first described by analyzing ESTs from *Phytophthora infestans* [58]. Also, 25 Crinkler-like sequences and 17 ELIs/ELLs were identified in a *P. sojae* EST data set [27]. Moreover, analysis of a haustorium specific EST library of *Melampsora lini*, a fungal rust pathogen of flax, has enabled identification of three secreted effector proteins that are recognized by specific R proteins [59].

It is notable that no putative function could be assigned for 108 unigenes corresponding to ∼55% of the predicted secreted proteins identified in our study, and therefore these sequences were classified as unknowns (Table 1). Further characterization of the secreted proteins of unknown function should reveal new aspects of the biology of oomycete pathogens as well as processes specific to *Hpa* biotrophy, since ∼47% of the unknowns (51 unigenes) appear to be *Hpa*-specific sequences.

From the set of secreted *Hpa* proteins with a predicted function we identified three Elicitin-like proteins. Although ELIs and ELLs have been described in *Phytophthora* and *Pythium* [49], [60], it is likely that the number of oomycete species in which these proteins are expressed will expand as more sequences become available. Indeed, our blast searches identified a homologue of HaELL3 in *Saprolegnia parasitica*. The cysteine spacing pattern allowed classification of *Phytophthora* ELIs and ELLs in different groups [49]. Interestingly, HaELL2 has the same pattern as some *Phytophthora* ELLs including INL1 (*P. infestans*) and SOL1A (*P. sojae*) that belong to ELL-1 group. The spacing pattern of HaELL1 and 3 was not observed in *Phytophthora*. Further analysis of elicitins from different oomycete species should establish if there are species-specific patterns of cysteine spacing. Two beta-elicitins (cryptogein and cinnamomin) have been shown to bind lipids [61], [62]. The majority of the residues of cryptogein that interact with ergosterol [63] are divergent in the three alpha HaELLs identified here as well as in *Phytophthora* ELLs [49]. Therefore, ELLs may not bind sterols and their function in the infection process remains unclear.

The 16 Cysteine-rich proteins identified in our survey belong to a specific class of effectors with a high relative number of cysteine residues (>5%) and no homology to other effectors [49], [64]. The HaCRs are a heterogeneous group since some proteins have homologues in other oomycetes, although the majority are *Hpa*-specific (Fig. 5; Table 2). So far no function could be assigned to the HaCR proteins based on known domains. Blast searches against the Emoy2 genome sequence revealed a high level of conservation of several HaCR proteins between these two isolates. Analysis of more *Hpa* isolates will reveal whether this degree of conservation is maintained in other isolates. Only HaCR7 was more divergent between the isolates Waco9 and Emoy2 (Table 2). It is worth noting that HaCR6 and 16 are not found in the Emoy2 genome, suggesting that a different combination of HaCR proteins might be present in each *Hpa* isolate.

The majority of the 18 RXLR candidate effectors identified in our study were *Hpa*-specific (Table 1). Five RXLR candidates comprise divergent protein variants in seven *Hpa* isolates (Fig. 6) and significant numbers of non-synonymous relative to synonymous nucleotide substitutions. These effector candidates add to a number of interesting *RXLR* genes that appear to be under diversifying selection [12] including the two *Hpa* effectors ATR1 and ATR13, known to that trigger ETI [11], [30].

We identified variations in the set of effectors produced by different *Hpa* isolates. Six Waco9 effectors (two HaCRs and four HaRXLRs) are not present in Emoy2, as determined by comparison with the Emoy2 genome sequence. Also, allele sequencing in seven *Hpa* isolates revealed that three RXLRs are so far only found in Waco9. The presence of an effector in some but not all isolates suggests that the gene has become lost during host-pathogen co-evolution to avoid triggering ETI. This has been described for effectors of different pathogens, including Avr4 from *Phytophthora infestans* [65] and Avr2 from *Cladosporium fulvum* [66], of which truncated forms that are not recognized by R proteins have been identified in several isolates [65]. However, delivery of RXLRs 12, 22 and 29 to leaves of 12 different Arabidopsis accessions using the EDV system did not reveal an obvious host cell death response which would typify ETI (unpublished data). This may be because the bacterial delivery system dampens recognition or doesn't permit full expression of ETI responses to specific *Hpa* effectors. Alternatively, isolate-specific effectors might affect colonization by different *Hpa* isolates by interfering mostly with host PAMP-triggered defenses. Our finding that RXLR29, an effector that is present only in the highly virulent *Hpa* isolate Waco9, is able to suppress PTI and enhance susceptibility to bacterial infection, supports this idea. The categorization of different effector protein types expressed by *Hpa* Waco9 during Arabidopsis leaf tissue colonization and before the developmental transition to asexual and sexual sporulation has allowed us to select effector candidates for further functional studies on interference with the host immune system. The activities and interactions of Waco9-derived effectors as well as the extensive allelic diversity of individual *RXLR* genes among *Hpa* isolates and oomycete pathogen species provides a rich source of material to trace the co-evolutionary history of this plant-biotroph system.

## Materials and Methods

### *Hpa* isolates and plant material

*Hpa* isolates used in this study were originally collected from Arabidopsis populations within the UK (isolates Cala2, Emwa1, Emco5, Emoy2, Hind2, Maks9, Noco2, Noks1) and the Netherlands (Waco9) [16], [18], [67], [68]. Arabidopsis plants were grown at 22°C with ∼75% relative humidity (RH) and a 10 h light period. For *Hpa* infections, conidiospore suspensions (5×10^4^ conidiospores.ml^−1^) were spray inoculated on 2-week-old Arabidopsis seedlings. Plants were allowed to dry for 1 h and kept at 100% RH for 24 h in a growth chamber with 10 h light at 16°C. Plants were then moved to ∼75% RH for an additional 4 to 6 days to delay asexual sporulation. *Hpa* growth on Arabidopsis leaves was visualized on whole-leaf mounts stained with trypan blue as described previously [67] and examined by differential-interference contrast microscopy.

### RNA isolation and cDNA library construction

For RNA isolations, infected leaf material was ground to a fine powder in liquid nitrogen. Total RNA of Waco9-infected Ws *eds1-1* plants was isolated using the RNeasy Plant Mini Kit (Qiagen), following manufacturer's instructions. Poly (A^+^) RNA was purified from 1.3 mg of total RNA using dynabeads oligo (dT)_25_ (Dynal Biotech). To eliminate rRNA contamination, the mRNA was purified twice. RNA concentrations were determined on a UV mini 1240 spectrophotometer (Shimadzu) and quality was assessed with the bioanalyzer 2100 using the RNA 6000 Nano Assay kit (Agilent Technologies). Poly (A^+^) RNA (5 µg) was used to construct a directional cDNA library in the λ-Zap vector (ZAP-cDNA synthesis, GigapackIII cloning kit, Agilent Technologies) following manufacturer's instructions. cDNA was synthesized containing EcoRI and XhoI sites at the 5′ and 3′ ends, respectively, allowing unidirectional cloning of cDNA. Size fractionation of the synthesized cDNA's was performed and 12 fractions were collected and precipitated with 100% ethanol. The pellet was resuspended in RNase-free water and verified on the Bioanalyzer 2100 using the DNA 7500 LabChip Kit (Agilent Technologies). Phagemid DNA was excised without library amplification. DNA isolations and sequencing were done by Macrogen (Korea). Sequencing reactions were performed from the 5′ end using a T3 promoter primer.

### Sequence processing and analysis

Base calling, quality clipping and vector screening were performed with pregap4, which is part of the Staden sequence analysis package [69]. ESTs were assembled into contigs using CAP3 [70]. To define sequence origins, the assembled sequences were queried (BLASTN, e-value cutoff 1 e-5, no low complexity filtering) [71] against versions 6, 8.3 and trace files of the *Hpa* genome [25], version 8 of the TAIR Arabidopsis genome [72], version 1 of the *Phytophthora infestans* [24], *P. ramorum* and *P. sojae* [23] genomes and NCBI non-redundant (nr) nucleotide database [73]. EST sequences were then split into subsets based on the best blast match. ESTs from *Ha* were submitted to dbEST (NCBI).

The most likely ORF of the *Hpa* set of sequences was identified by translating the assembled EST sequences and singletons in 3 positive frames and selecting the longest ORF. Protein predictions shorter than 10 amino acids were discarded. For selection of the secreted proteins, all protein models were trimmed to start with a methionine. Signal peptide predictions were performed by SignalP version 3.0 [43], [44], using both the neural network method and the hidden markov model methods at default cutoffs. Sequences with predicted transmembrane helices downstream of the signal peptide determined by TMHMM version 2 (http://www.cbs.dtu.dk/services/TMHMM/) [74] were discarded. Putative functions were assigned by domain composition after scanning the sequences against Pfam [45] using the gathering threshold as cut-off. Additionally, functions were manually assigned based on homology derived by similarity matches to NCBI nr (using blast), amino acid composition or presence of an RLXR, RXLQ or RXLG motif after the signal peptide cleavage site (only considering proteins with 40 or more amino acids after the RXL sequence). The number of effectors encoded in the *Hpa* genome was either taken from the *Hpa* genome paper [25], determined by Pfam searches, or by manual annotation (e.g. for the number of cysteine residues). For the elicitins, the pI was determined at http://www3.embl.de/cgi/pi-wrapper.pl. GPI anchor sites were predicted by the program big-PI Plant Predictor [75] and potential sites for O-glycosylation were predicted by NetOGlyc 3.1 [76]. Psort prediction (http://psort.hgc.jp/) [77] was used to identify putative nuclear localization signals in RXLR proteins. Sequences of the *HaELL*, *HaCR* and *HaRXLR* genes were submitted to GenBank (accession numbers JF800099-JF800135).

### Identification of *Hpa*- and isolate Waco9-specific sequences

For identification of secreted proteins specific to *Hpa* or to isolate Waco9, similarity searches were conducted with BLASTP and TBLASTN with disabled low complexity filtering and a fixed database size of 1.000.000 [70]. Sequences were queried against a local NCBI nr protein and nucleotide databases (downloaded 23^rd^ June 2010) and a set of three oomycete genome sequences (*P. infestans* version 1 [24], *P. sojae* version 2, and *P. ramorum* version 2 [23].Sequences with no significant similarity against other organisms (*Hpa* sequences were excluded; e-value cutoff 1e^−5^; covered for >75% to exclude hits with only local similarity) were selected and defined as *Hpa*-specific sequences. The predicted proteome and the genome assembly of *Hpa* isolate Emoy2 (versions 6.0 and 8.3) was searched to identify sequences potentially specific for isolate Waco9. All sequences with a significant hit (e-value cutoff 1e^−10^) and an identity ratio of >80% ((identity% of alignment*length alignment)/length query) [26] were filtered. If two Waco9 sequences had the same Emoy2 sequence as a best hit, we excluded the better hit and retained the other one. The remaining sequences were compared manually to the *Hpa* Emoy2 trace files that also contain reads not present in genome assembly. Sequences without significant blast hits were defined as Waco9-specific.

### *Hpa* allele sequences and analysis

To sequence the RXLR alleles of *Hpa* isolates Cala2, Emco5, Emoy2, Hind2, Maks9 and Noks1, genomic DNA was isolated from *Hpa*-infected Ws *eds1-1* leaves using DNeasy Plant Mini Kit (Qiagen). Primer pairs (Table S1) flanking or within the coding sequence were designed from Waco9 assembled sequences and used to amplify alleles of the *Hpa* isolates from genomic DNA. If a single amplification product was obtained, the PCR product was sequenced directly using the amplification primers. If multiple PCR fragments were amplified from a single isolate these were individually purified from gel using NucleoSpin Extract II (Machery-Nagel) and sequenced. Obtained sequences that were not readable due to amplification of different products of the same size, Phusion (Finnzymes)-amplified PCR products were cloned in pENTR/D-TOPO (Invitrogen) and sequenced. In cases where the obtained sequences were not full-length (3′or 5′ sequences missing), new primers were designed based on the Emoy2 genome sequence (Versions 6 and 8.3). When no PCR product was amplified, new reactions were performed using up to three new primer sets.

The RXLR DNA and predicted protein sequences were analyzed using Mega4 software [78]. Alignments of the obtained allele sequences (protein level) were performed using the ClustalW option [79] and manually edited. The number of variable sites was analyzed by the Sequence Data Explorer tool. Alignments of the allele sequences (nucleotide level) were used to calculate the rate of non-synonymous (dN) and synonymous (dS) substitutions. The rates of substitutions were calculated using the Nei and Gojobori's method [80], as implemented in the MEGA4 program. Standard error was determined by 500 bootstrap replications. The null hypothesis of no selection (H0: dN = dS) versus the positive selection hypothesis (H1: dN > dS) were tested using the Z-test: Z = (dN-dS)/ √(Var(dS)+Var(dN)).

### RXLR29 delivery by Pseudomonas syringae

The coding sequences of *Hpa* Waco9 RXLR29 (without signal peptide) and YFP were amplified by PCR and cloned in pENTR/D-TOPO (Invitrogen) using primers: CACCATGGAGGTGGTCCTGATC (forward) and TTACTTGCCAGGACGCGC (reverse) for RXLR29 and CACCATGGTGAGCAAGGGCGAGGAGCTGTTC (forward) and AGTCTAGAGCTCTTACTTGTACAGCTCGTCCATGC (reverse) for YFP. Following Gateway cloning procedures these genes were cloned into pEDV6, a variant of the previously described pEDV3 [42] that has a gateway cassette instead of a multiple cloning site (kindly provided by K. Sohn, G. Fabro and J. Jones, Sainsbury Laboratory, Norwich, UK). Plasmids were mobilized from *E. coli* DH5α to *Pst* DC3000ΔCEL or *Pst* DC3000-LUX, which has stable chromosomal integration of the luxCDABE operon from *Photorhabdus luminescens* [81], by standard triparental mating using *E. coli* HB101 (pRK2013) as a helper strain. *Pst* DC3000ΔCEL-ATR13 (Emco5) and *Pst* DC3000-LUX-ATR13 (Emco5) were kindly provided by G. Fabro and J. Jones.

### Callose staining and microscopic analysis

Leaves of 5-week-old Arabidopsis accession Col-0 plants were hand-infiltrated with 1×10^8^ cfu/ml *Pst* DC3000ΔCEL suspensions. A total of ∼48 leaf samples were taken for callose staining 12–14 h after infiltration. Leaves were cleared with 100% ethanol, re-hydrated and stained with aniline blue (0.05% in phosphate buffer pH8.0) for 24 h. Samples were analyzed with an Olympus AX70 Microscope using an UV filter. Callose spots were counted using the ImageJ software (http://rsb.info.nih.gov/ij/) [82].

### Bacterial growth assay

Leaves of 5-week-old Arabidopsis accession Col-0 were hand-infiltrated with a bacterial inoculum of 5×10^5^ cfu/ml. Initially, *Pst*DC3000-LUX was used for assessment of bacterial growth by measuring increased luciferase activity as previously published [81]. For colony counting, infected leaves were collected at 0 and 3 days after bacterial infiltration. A total of 12 leaves divided into three biological replicates were harvested per sample and time point. Bacterial growth was measured by grinding infected leaves and plating serial dilutions on solid KB medium with appropriate antibiotics. Similar results were obtained in two independent experiements.

## Supporting Information

Figure S1 **Growth of** ***Hpa*** **Emoy2 and Waco9 isolates in Arabidopsis.** The level of colonization of cotyledons of 2-week-old Arabidopsis Ws *eds1-1* seedlings infected with *Hpa* isolates Emoy2 (A,B) and Waco9 (C,D) at 7 dpi is visualized by trypan blue-staining and light microspcopy.
(EPS)

Figure S2 **Determination of** ***Hpa*** **rRNA peaks on the Bioanalyzer 2100.** Total RNA isolated from *Hpa* conidiospores and Arabidopsis leaves was mixed in different proportions to allow identification of the corresponding rRNA peaks of *Hpa* and Arabidopsis in bioanalyzer profiles. Peak sizes correlated with the relative amount of plant and pathogen total RNA.
(EPS)

Figure S3 **Schematic representation of the** ***Hpa*** **Crinkler identified in the Waco9 cDNA library.** The signal peptide (SP), the variable CRN motif (LYVAK) and the C-terminal domain (C) are shown.
(EPS)

Figure S4 **Multiple alignment of RXLR29 sequences from 7** ***Hpa*** **isolates.** Insertion/deletion sites are indicated in yellow, stop codons are indicated in red.
(EPS)

Table S1 **Primers used for RXLR allele sequencing.**
(XLS)

Table S2 **Enrichment of protein class members in the EST project compared to the genome-wide occurrence.** A hypergeometric propability value <0.05 in the Enriched column indicates a significantly higher than average occurrence in the EST set of genes, e.g. all predicted secreted proteins (total).
(XLS)

Table S3 **Functional classification of 90 unigenes based on Pfam domain predictions, BLASTX and manual annotation.**
(XLS)
